# A White Plaque, Associated with Genomic Deletion, Derived from M13KE-Based Peptide Library Is Enriched in a Target-Unrelated Manner during Phage Display Biopanning Due to Propagation Advantage

**DOI:** 10.3390/ijms23063308

**Published:** 2022-03-18

**Authors:** Danna Kamstrup Sell, Ane Beth Sloth, Babak Bakhshinejad, Andreas Kjaer

**Affiliations:** Department of Clinical Physiology and Nuclear Medicine & Cluster for Molecular Imaging, Copenhagen University Hospital-Rigshospitalet & Department of Biomedical Sciences, University of Copenhagen, 2200 Copenhagen, Denmark; danna@sund.ku.dk (D.K.S.); anebeth@sund.ku.dk (A.B.S.); babak.bakhshinejad@sund.ku.dk (B.B.)

**Keywords:** biopanning, competitive propagation, genomic deletion, lacZα sequence, M13KE, phage display, Ph.D.^TM^-7 peptide library, propagation-related TUP, white plaque

## Abstract

The nonspecific enrichment of target-unrelated peptides during biopanning remains a major drawback for phage display technology. The commercial Ph.D.^TM^-7 phage display library is used extensively for peptide discovery. This library is based on the M13KE vector, which carries the lacZα sequence, leading to the formation of blue plaques on IPTG-X-gal agar plates. In the current study, we report the isolation of a fast-propagating white clone (displaying WSLGYTG peptide) identified through screening against a recombinant protein. Sanger sequencing demonstrated that white plaques are not contamination from environmental M13-like phages, but derive from the library itself. Whole genome sequencing revealed that the white color of the plaques results from a large 827-nucleotide genomic deletion. The phenotypic characterization of propagation capacity through plaque count- and NGS-based competitive propagation assay supported the higher propagation rate of Ph-WSLGYTG clone compared with the library. According to our data, white plaques are likely to arise endogenously in Ph.D. libraries due to mutations in the M13KE genome and should not always be viewed as exogenous contamination. Our findings also led to the conclusion that the deletion observed here might be an ancestral mutation already present in the naïve library, which causes target-unrelated nonspecific enrichment of white clone during biopanning due to propagation advantage.

## 1. Introduction

Phage display is a powerful and popular high-throughput selection technique to identify peptide ligands with specific binding to physiologically relevant protein and non-protein targets. In phage display, the DNA encoding an exogenous (poly)peptide is cloned into a specific site in the phage genome that encodes one of the phage coat proteins. After infection of the host bacterium and expression of phage genes, the guest (poly)peptide encoded by exogenous DNA is expressed as part of the relevant coat protein while its encoding sequence is located within the phage genome. Thus, the method exploits the direct physical link between a (poly)peptide expressed on the phage outer surface (phenotype) and its encoding DNA in the genome (genotype). This phenotype-genotype link serves as the backbone of phage display technology and allows for sequence determination of selected ligands displayed on the surface of phage virions. [1,2]. In the commercially available and commonly used libraries, such as those provided by New England Biolabs (NEB), a wide diversity of randomized peptide sequences are expressed as fusions to the pIII coat protein of the phage vector M13KE, and the naïve library is said to consist of around 10^9^ unique peptide sequences. NEB has manufactured three different random peptide libraries, including linear heptapeptide (Ph.D.^TM^-7), cyclic heptapeptide (Ph.D.^TM^-C7C), and linear dodecapeptide (Ph.D.^TM^-12) libraries. In the cyclic library, the displayed peptide is flanked by a pair of cysteine residues, which form disulfide bonds during phage assembly. The complexity of heptapeptide libraries can represent approximately all of the possible 7-mer peptide sequences but only a minor fraction of all possible 12-mer peptide sequences. In all libraries, the first amino acid of the peptide-pIII fusion is the first randomized position, and there is a short linker (Gly-Gly-Gly-Ser) between the displayed peptide and pIII [3,4]. As the phage vector used in this library is a simple M13mp19 derivative, it carries the lacZα gene, allowing for blue-white screening and enabling the formation of blue plaques on IPTG-X-gal-containing plates. According to the manual provided by the manufacturer, the emergence of white plaques indicates contamination of the library with environmental M13-like phages during panning and amplification [3], which can be devastating for the selection process.

The phage display library screening is performed through biopanning, in which multiple rounds of selection and amplification are applied to enrich the pool of phage clones displaying peptides with high binding affinity towards the target of interest [5]. Although phage display has offered enormous potential in the identification of a variety of target-avid peptides, an inevitable consequence of biopanning is the emergence of false-positive peptides. Peptides that emerge in rounds of biopanning without specific binding affinity towards the target are termed target-unrelated peptides (TUPs) [6]. Generally, these peptides are classified as either selection-related (Sr-TUPs) or propagation-related (Pr-TUPs). Sr-TUPs arise as a result of binding to one of the components of the selection system other than the target [6]. Pr-TUPs emerge because the phage library is based on a biological system where genetic changes, like point mutations, may occur. Such variations can lead some clones to gain a biological propagation advantage compared to other clones in the library. Pr-TUPs are therefore recognized as peptides coincidentally expressed on phage clones with faster propagation rates [7,8]. To date, a number of fast-propagating phage clones displaying different peptide sequences have been identified, and their higher propagation has been attributed to various genetic alterations [7,9]. 

The identification and removal of TUPs arising in biopanning output is of utmost importance since they can be mistaken as target-related peptides (TRPs) and misguide further downstream characterization of isolated peptides. Discrimination between false-positive TUPs and true target binders makes a significant contribution to the integrity of phage display selection and paves the way for translation of biopanning findings into the development of diagnostics and therapeutics. In recent years, some experimental [8,9] and in silico [10,11] methods have been developed for TUP identification. These TUP discovery approaches have provided novel insights into the selection procedure and expanded the repertoire of non-specific binding peptides isolated in phage display. Here, we report the isolation of a non-target binding fast-propagating phage clone (displaying the peptide WSLGYTG) through biopanning of Ph.D.^TM^-7 (a NEB-made 7-mer random peptide library) on recombinant mouse CD4 protein. Instead of blue-colored plaques, this clone produced white plaques. Whole genome sequencing (WSG) uncovered a large deletion, removing the lacZα sequence, in the genome of this phage, and a competitive propagation assay revealed its high propagation rate. Our findings suggest that WSLGYTG-displaying white clone has been enriched in biopanning due to propagation advantage, not true binding affinity to the target.

## 2. Results

### 2.1. White Plaques Appeared in Biopanning Output Originate from the Library

**Figure 1:**
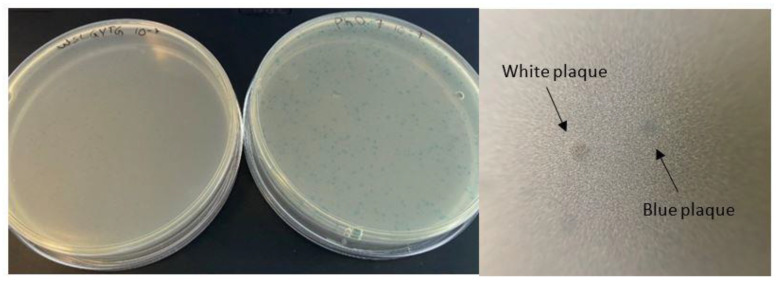
Plaques from WSLGYTG-displaying phage (left plate) and Ph.D.^TM^-7 peptide library (right plate) plated on separate plates and a close-up of a white and a blue plaque. WSLGYTG-displaying clone produces white plaques, whereas library clones produce blue plaques.

After three rounds of biopanning of the Ph.D.^TM^-7 library on recombinant mouse CD4, 45 plaques were picked out for sequencing to recognize displayed peptides. The phage clone displaying the WSLGYTG peptide was identified as the most enriched clone (4 clones, corresponding to 8.9% of phage clones) and was therefore subjected to downstream characterization. However, upon plating the amplified WSLGYTG-displaying clone for titration, it was found that the color of the plaques appearing on the plate was white (Figure 1). The cloning vector WT-M13KE used for the construction of the Ph.D.^TM^-7 peptide library differs from wild-type environmental M13 (WT-M13) phages by the insertion of a lacZα-containing sequence into the vicinity of the origin of replication (*Ori*) that leads library phages to generate blue plaques in the presence of IPTG (an inducer of the *lac* operon in the *E. coli* genome) and X-gal (enzyme substrate). Therefore, the emergence of white plaques was first assumed to arise from the contamination of WSLGYTG-displaying clone stock with white plaque-producing environmental phages. 

**Figure 2:**
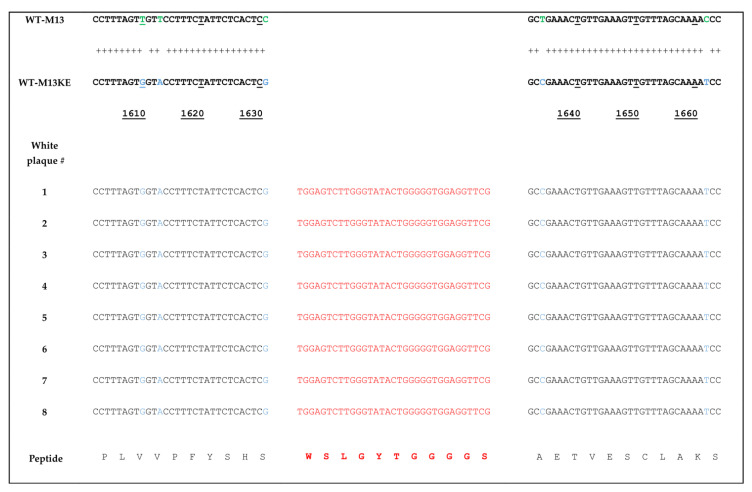
DNA sequence alignment between a fraction of the genomes of WT-M13 (at the top), WT-M13KE (in the middle) and eight isolated white plaques (at the bottom). All eight clones show evidence of arising from the library as they contain nucleotides (marked in blue) that differentiate them from the corresponding nucleotides in WT-M13 (marked in green). Additionally, all the eight white clones express the peptide WSLGYTG and the linker GGGS (marked in red), which are not present in the genomes of both WT-M13 and WT-M13KE. The nucleotide numbers refer to WT-M13KE genome. Compared to WT-M13, the nucleotide numbers are lower by 1 in WT-M13KE due to the deletion of T at position 1565. The alignment analysis was performed in SnapGene software (from Insightful Science; available at snapgene.com).

Several white plaques were picked out and sequenced. As indicated in Figure 2, the genomic sequence of white plaques is identical to WT-M13KE and shows a difference from WT-M13. The genome of library-derived WT-M13KE has multiple nucleotide differences in comparison with the genome of environment-derived WT-M13. These nucleotide differences serve as a sequence signature to distinguish WT-M13KE from WT-M13. We sequenced the genomes of eight white plaques (nucleotides 1130 to 1680). This genomic part of the M13 phage contains five nucleotide differences between WT-M13 and WT-M13KE, highlighted in green and blue colors in the upper part of Figure 2 (G at position 1610, A at position 1613, G at position 1631, C at position 1634, and T at position 1662). The presence of these nucleotides in white plaques indicates that viral plaques derive from the library, not the environment. Additionally, all the sequenced white clones display the WSLGYTG peptide (marked in red), which is not present in the genome of WT-M13KE (the reference genome not displaying any peptide). 

### 2.2. WSLGYTG-Displaying White Clone Does Not Show Significant Binding to the Target Protein

**Figure 3:**
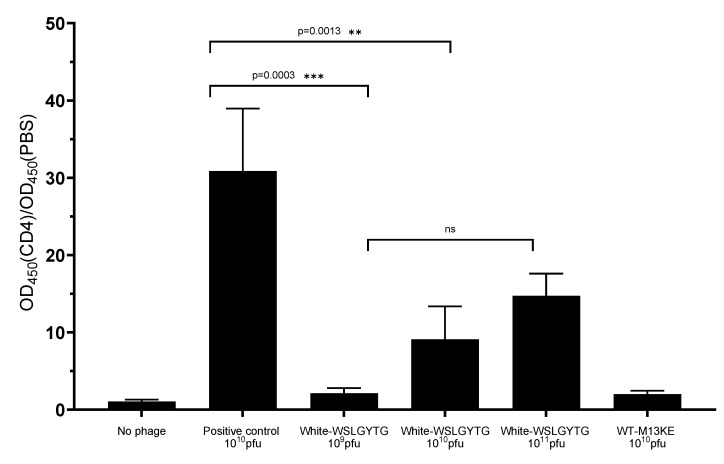
Evaluation of WSLGYTG-displaying white clone binding to the target protein mCD4. Target-coated wells (0, 5 µg/well) were incubated with phage clones (White-WSLGYTG, WT-M13KE, positive control, and no phage). The plate was incubated with anti-M13 antibody conjugated with HRP and color development was achieved by adding TMB and quenching with H_2_SO_4_. The absolute absorbance values (450 nm) were reference subtracted (540 nm) and binding ratio was determined through dividing clone binding by CD4-coated/PBS-coated wells (OD450 (CD4)/OD450 (PBS)). The white-WSLGYTG clone does not show significant binding to CD4 once compared with positive control. PBS: Phosphate buffered saline (no target), WT-M13KE: Ph.D.^TM^-7 library clone without displayed peptide, ns: non-significant.

To investigate the binding of WSLGYTG-displaying white clone to the target protein, enzyme-linked immunosorbent assay (ELISA) was performed, and the binding ratio was calculated for each phage sample based on the absorbance from CD4-coated wells divided by the absorbance from PBS-coated wells (Figure 3). The following two different negative controls were included: no phage (to account for nonspecific binding of anti-M13 antibody in the target-coated well) and WT-M13KE as a Ph.D.^TM^-7 library clone that does not display any peptide on its surface (to account for the nonspecific binding of the phage particle itself to the target). Moreover, a positive control was included in ELISA that was a clone already shown to have high binding affinity for the target. To have a more accurate evaluation of the target binding of the WSLGYTG-displaying white clone, three different titers of this clone (10^9^, 10^10^, and 10^11^ pfu) were used in ELISA. 

According to our results, the white clone indicated a significantly lower target binding than the positive control in the same phage titer (10^9^ pfu). Consistent with this, the mean ratio of positive control (average ratio of 30) was significantly higher than the white clone (average ratio of 9.1) (*p* = 0.0003). Moreover, the white clone did show a significantly higher target binding than WT-M13KE (negative control). Input phage titer is an important factor for color development in phage ELISA. To have a more accurate evaluation of the target binding capacity of the WSLGYTG-displaying white clone, the binding of this phage at a higher titer (10^11^ pfu) was compared with a positive control (10^10^ pfu). Interestingly, even with a 10-fold increase in the input titer of white clone (100 billion virions of white clone vs. 10 billion virions of positive control), the target binding of this phage was still significantly lower than the positive control (*p* = 0.0013). The results of phage ELISA provide support for the notion that although the clone displaying WSLGYTG peptide has been enriched and selected in biopanning, it is not a true target-specific binder.

### 2.3. Whole Genome Sequencing of White Clone Indicates a Large Deletion in the Phage Genome

**Figure 4:**
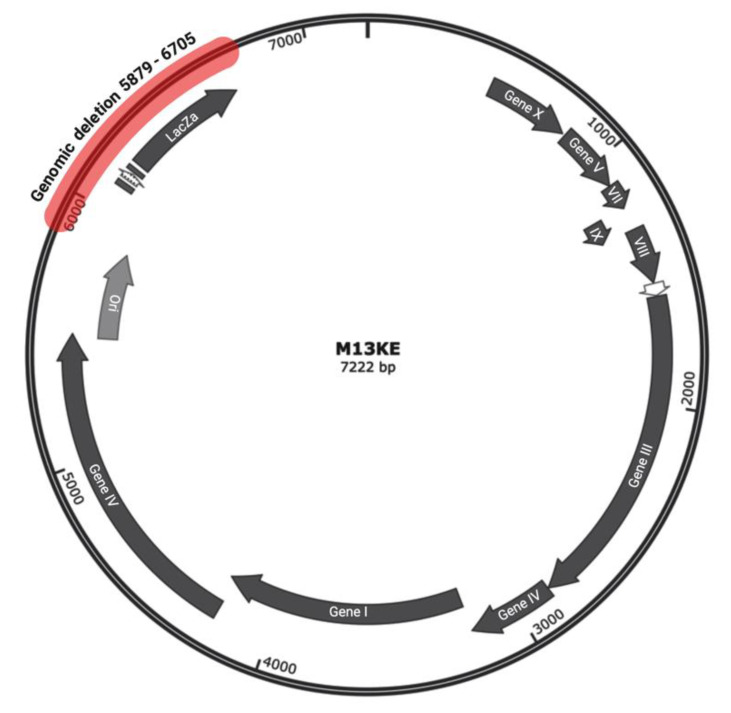
Schematic representation M13KE genome indicating large intergenic deletion (red) revealed by whole genome sequencing. The deletion was detected in positions 5879–6705 (827 nucleotides), causing deletion of the segment encoding lacZα gene. The M13KE genomic information was downloaded from New England Biolabs repository [12] and the figure was prepared using SnapGene and BioRender.

Whole genome sequencing (WGS) analysis was conducted to gain insight into any probable alterations in the genome of the white clone. The results of WGS revealed a large deletion in the genome from nucleotide 5879 to nucleotide 6705 (numbering refers to M13KE). This deletion includes almost the entire intergenic region between *Ori* and lacZα (except the first 11 nucleotides immediately after *Ori*), the complete lacZα gene, and 10 nucleotides immediately downstream of the lacZα gene (Figure 4).

### 2.4. Competitive Propagation Assay (CPA) Reveals a High Propagation Rate for White Clone

The observation of no significant binding affinity for the white-Ph-WSLGYTG clone towards CD4 in phage ELISA inspired us to advance the hypothesis that this phage is likely to be enriched during library selection in a nonspecific manner and represents the potential to display a TUP. Another insightful finding that strengthened our original proposition was the results of WGS analysis based on which white-Ph-WSLGYTG clone had a large deletion, leading to a dramatic reduction in genome size (the loss of about 11.5 percent of the total genome). These different lines of evidence prompted us to investigate whether this clone possesses a particular propagation advantage compared with the naïve Ph.D.^TM^-7 library, as the library contains a huge variety of phages with a range of propagation rates. To this end, competitive propagation assay was conducted in which equal titers of white-Ph-WSLGYTG and library virions were inoculated together in an early log E.coli ER2738 culture. At three time points, samples were assessed with respect to white/blue plaque titer by plate count method and NGS analysis.

In the blue/white plaque count titration (Table 1), we observed a comparably equal titer of blue and white plaques immediately after mixing (t = 0). During the amplification period, the titers of both white and blue clones were increased. However, titer enhancement was dramatically higher for white clones compared with blue ones.

**Figure 5:**
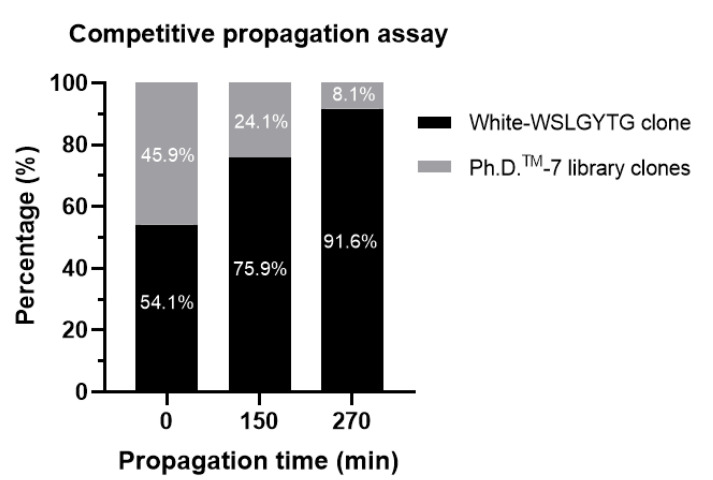
NGS-based competitive propagation assay. Equal titers of white-WSLGYTG clone and Ph.D.^TM^-7 library were inoculated in early log E.coli culture and amplified for 4.5 h. At three time points, samples were withdrawn and the percentage of WSLGYTG-displaying clone compared to random peptide library clones was calculated based on NGS data. A substantially higher propagation rate was found for WSLGYTG clone compared to the library (representing an average propagation rate of the clones in the library).

We also performed competitive propagation assay based on NGS. As white plaques displayed WSLGYTG, this assay was performed to determine the number of WSLGYTG-displaying virions in the phage pool. Table 2 contains information about the number of reads acquired and analyzed in the NGS-based CPA. The sequencing quality was optimal, and only a low percentage of reads were removed after filtering. Clean reads were used for the analysis of CPA results. Immediately after mixing, a sample was withdrawn (t = 0) and phages displaying the peptide WSLGYTG comprised around half (54.1%) of the phage virions present in the culture. After 150 min, WSLGYTG clone was able to take over the majority of the growing culture (75.9%) and, after 270 min became immensely predominated in the culture (comprising 91.6% of the total number of phage virions) (Figure 5). The findings of the competitive propagation assay confirmed our hypothesis, revealing the fact that the white-Ph-WSLGYTG clone has a significantly higher propagation rate in comparison with the average propagation rate of phage clones in the library. On this basis, WSLGYTG is characterized as a Pr-TUP displayed on the surface of a fast-propagating phage clone that produces white plaques.

## 3. Discussion

Phage display is a potent strategy for the discovery of target-specific ligands. The high throughput nature of this methodology enables researchers to screen very large and diverse libraries for the identification of clinically relevant peptides [13,14]. Although many successful selections have been described in the literature, biological bias is an inherent feature of phage display libraries. The undesirable isolation of TUPs without true binding affinity toward the target remains a major bottleneck for this technology. Pr-TUPs, one of the most important categories of phage display-selected false-positive binders, are coincidentally displayed on viruses that propagate faster than other clones of the library [8], and their target-independent enrichment has the potential to negatively impact the selection output. Distinguishing TUPs from target-specific peptides and reporting propagation-related predominating phage clones will facilitate the discovery of true target binders, illuminating the dark side of biopanning. In the current work, we report the isolation of a fast-propagating phage clone displaying the peptide WSLGYTG, which was enriched through screening of the commercial Ph.D.^TM^-7 phage display library against recombinant mouse CD4 protein. A literature search revealed that this peptide sequence has already been isolated in biopanning on two other targets, including human recombinant CD3_Ɛ_ protein (the epsilon chain of T-cell surface glycoprotein CD3) [15] and protective and non-protective anti-*Borrelia burgdorferi* sera of New Zealand White (NZW) rabbits [16]. However, the chance of obtaining a true binder on different targets in biopanning of a library containing billions of peptides is very unlikely, and the peptide might be a nonspecific ligand. In line with this, the results of phage ELISA uncovered that the Ph-WSLGYTG clone does not show significant binding to the target protein (Figure 3). Another observation that raised our interest in further investigation of the Ph-WSLGYTG clone was the white color of the plaques produced by this clone (Figure 1). According to the manufacturer’s instructions [3], the most likely explanation for the emergence of white plaques is that the phage pool has become contaminated with environmental M13-like phages during panning and amplification. Nonetheless, our findings on the genomic analysis of randomly chosen white plaques (Figure 2) present a stark contrast to this notion and evidently demonstrate that all the white plaques in our work originated from the library, with no environmental contamination. There is also one previous report on the identification of library-derived white plaques from M13KE-based libraries [17]. Our WGS analysis of the Ph-WSLGYTG indicated that a large genomic deletion, which removes the entire lacZα gene (from 6216 to 6695), causes the white color of plaques. The deletion covers nucleotides 5879 to 6705 (a total of 827 nucleotides) (Figure 4) in the phage genome, which includes a major part of the intergenic region between gIV and gII, goes 10 nucleotides downstream of the lacZα gene and ends 115 nucleotides before gII [12]. Interestingly, the deletion starts 11 nucleotides downstream of the origin of replication (5487 to 5867), thus not impacting *Ori*. *Ori* is essential for M13 phage DNA replication and is highly sensitive to mutations [18,19]. In the library vector M13KE, a large 816 nucleotide fragment is inserted into the intergenic region near *Ori* [20]. The proximity of this large insert to *Ori* gives rise to a significant reduction in the propagation capacity of the M13KE phage compared with wild-type M13. Therefore, the genomic deletion observed in our work is suggested to act as a compensatory mechanism for the replication defect of M13KE. However, the degree of deletion in terms of size and position may vary. Consistent with our finding, Smith et al. [7] have already reported the occurrence of large genomic changes (rearrangement) in compensation for reduced propagation in fd-based phage display libraries. 

To perform phenotypic characterization, the propagation rate of white-Ph-WSLGYTG was compared with library (containing phage clones with a range of propagation rates) via CPA. In contrast to time course analysis for phage amplification in which different monoclonal phage pools are compared individually [8], a polyclonal phage pool is investigated in CPA and, thus, evolutionary competition among virions serves as a basis for comparison of the amplification profiles of different phage pools. Based on competition in a polyclonal phage pool, CPA mimics library construction and its subsequent selection through biopanning. Since the plaque count approach solely assesses blue/white phenotypes (not displayed peptides), we also sequenced the CPA phage pool to assess the frequency of WSLGYTG-displaying phages. Since plaque count is highly prone to bias due to the small sample size (counting less than 100 white and blue plaques), we conducted CPA using NGS to allow for a strikingly larger number of virions to be analyzed (around 10^6^ according to the MiSeq platform used in our work (Table 2 and Figure 5). Mapping a larger fraction of the phage pool yields in-depth and more detailed insights into the trend of sequence enrichment during the amplification period. Both enhancing white to blue ratios/percentages over time in plaque count-based CPA (Table 1) and a dramatically increasing percentage of WSLGYTG-displaying clones in NGS-based CPA (Figure 5) demonstrated a clear dominance of white-Ph-WSLGYTG in the final phage pool, supporting the faster propagation rate of this phage over other library clones. The findings of CPA highlight that persistent occurrence in selection rounds and the final isolation of white-Ph-WSLGYTG clone at the end of biopanning is not due to specific target binding but results from increased evolutionary fitness for propagation associated with genomic deletion. It might be speculated that a smaller genome (over 10% reduction in genome size), removal of coding sequences (like lacZα), and increased *Ori* function all lower the energy cost of transcription and translation, allowing the phage to amplify faster. One possible explanation for the nonspecific enrichment of white clones and their appearance as one of our most abundant peptides is the diversity collapse that happens during biopanning. Therefore, the phage pool in biopanning is remarkably less diverse and, thus, contains fewer competitors than the library used in CPA. This can lead to the highly efficient enrichment of fast-propagating clones during amplification steps conducted between the rounds of biopanning. 

The M13 phage virion is a flexible rod-shaped nucleoprotein filament about 6 nm in diameter and about 900 nm in length [21,22]. This filamentous structure consists of a single-stranded circular DNA that is surrounded by a protein coat called a capsid. The capsid is a symmetric array of around 2700 copies of α-helical pVIII, which is known as the major coat protein, and a few copies (three to five) of each of the four minor coat proteins at the two ends of the virion (pIII, pVI at one end, and pVII, pIX at the other end) [22,23,24]. Subunits of pVIII interact with each other and with the viral ssDNA to generate structurally stable and functionally viable phage virions. It is known that M13 has a variable length and the virion length is dictated by the size (number of nucleotides) and conformation of the packaged DNA. Therefore, the number of nucleotides in the genome ascertains the exact number of pVIII subunits (as the determinant of virion length), and thus, the ratio of nucleotides per coat protein subunit (n/s ratio) [25]. It has been indicated that although the length of a wild-type virion is about 900 nm, shorter virions can also be assembled [21,26]. The smaller genome is packaged by a lower number of pVIII proteins, leading to the formation of a smaller virion. As mentioned earlier, the genome of the white-Ph-WSLGYTG clone is over 10% shorter than the genome of the blue plaque-producing clones in the library. Therefore, the white clone is expected to have shorter virions and fewer pVIII subunits. A lower copy number of pVIII might help the phage propagate faster. Electron microscopy (EM) techniques have already been used to characterize filamentous phages such as M13, providing detailed structural information with respect to the geometry and morphology of these phages as well as the structures of their different proteins [27,28,29,30,31,32,33]. Based on this, the use of EM holds potential to obtain insights into the length and structure of white-Ph-WSLGYTG. This kind of structural analysis can help us to make a comparison between the dimensions of white and blue virions as well as understand whether there is a correlation among virion length, the number of pVIII subunits, and propagation advantage. 

In silico tools such as Biopanning Data Bank (BDB) and Scanner and Reporter of Target-Unrelated Peptides (SAROTUP) have been developed for cleaning biopanning data. The BDB contains already reported biopanning results from peer-reviewed papers and is the most comprehensive database for the analysis of peptides obtained through the screening of phage display libraries [10,11]. SAROTUP was developed to identify TUPs in phage display experiments. PhD7Faster version 2.0, as part of SAROTUP, is a machine learning-based predictor made using NGS data of amplified Ph.D.^TM^-7 library [34]. It can predict if a peptide from this library is a Pr-TUP. Once we searched for WSLGYTG in BDB, no hits were found. Although WSLGYTG has already been reported in biopanning data from the Ph.D.^TM^-7 library, it has not been deposited into BDB yet. This reflects the fact that these repositories might not cover all the peptide sequences found and reported in phage display studies. Furthermore, inconsistency exists between our experimental findings and PhD7Faster in silico predictions on the propagation capacity of the WSLGYTG-displaying white clone. PhD7Faster predicts a low probability value (0.1) for the propagation rate, whereas our experimental data provide strong support for the propagation advantage of white-Ph-WSLGYTG. Although a high probability of propagation advantage predicted by PhD7Faster most likely results from the genotype of the pertinent phage clone, the tool solely identifies a clone as fast propagating based on the sequence of the displayed peptide. As a matter of fact, PhD7Faster forges a connection between genotypic characteristics and the displayed peptide. The peptide merely acts as a guide to identify a clone as fast-propagating and does not directly take genotypic characteristics into consideration. This means that PhD7Faster cannot make a distinction between the white clone displaying WSLGYTG in our work and the already reported CDɛ-binding blue clone displaying WSLGYTG [15]. The two clones display the same peptide but possess different genotypes and plaque colors. This leads PhD7Faster to recognize both virions as slow-propagating clones (propagation probability value = 0.1). In this manner, the propagation-enhancing genomic deletion in the white clone is neglected. Another drawback of propagation prediction tools, such as PhD7Faster, is their lot-dependency. Different lots of the same library type are very likely to contain phages displaying the same peptide but having different genotypes. This can simply lead to contradictions between experimental data and in silico analyses of phage propagation, underscoring that bioinformatic tools are solely in silico predictors and should be used with caution. 

The deletion observed in our work is the same as reported by Zygiel et al. [17]. They found the deletion in three different non-commercial libraries (ACX_4_CX_2_, ACX_5_CX_1_, and ACX_6_C) constructed using the M13KE vector. The observation of the same large deletion in different M13KE-based libraries in two different studies poses an important question about the temporal occurrence of this genomic alteration. Two scenarios exist about the origin of white plaques. Based on the first scenario, the deletion happened during amplification steps between rounds of biopanning and the clone displaying the WSLGYTG peptide existed in the naïve library as a blue plaque-producing virus. Zygiel et al. suggested a possible mutational hotspot in the phage genome as an explanation for the emergence of white plaques in three different libraries. According to this standpoint, independent deletion events have occurred separately in different M13KE vector molecules. This notion contradicts the fact that indels are generally infrequent compared to point mutations [35]. This is more pronounced for larger indels, and consequently, it is very unlikely that such a large (827 nucleotide) deletion recurs in different libraries. On the other hand, the frequent occurrence of such mutational events might offer some clues about the instability of the M13KE genome, posing a limitation to its use in the construction of phage display libraries. The second scenario proposes that the deletion was already present as a very minor portion of the M13KE molecules initially employed to generate the libraries or arose during the amplification step of library construction. In the procedure of library construction, the ligation of the displayed peptide-encoding oligonucleotide pool into M13KE is followed by transformation into the host bacterium and intracellular vector amplification. Owing to the propagation advantage, the deletion mutant clone in the naïve library gets the chance to become enriched and increases its copy number during subsequent amplification steps between biopanning rounds, leading to its propagation-related target-unrelated enrichment in the recovered phage pool from the last round of selection. In this scenario, an ancestral mutation exists in some M13KE molecules of the naïve library that is passed down to all the libraries made from vector stock containing the mutation. In this case, the different molecules of the mutant vector can display different peptide sequences. This has been observed for the deletion mutant white clone which displays WSLGYTG (from the Ph.D.^TM^-7 library) in our work, whereas it displays ACSYKACWV, ACHYAPCRS, and ACLALACRT (from the ACX_4_CX_2_ library), ACRSAGTCP and ACQYAKLCA (from the ACX_5_CX_1_ library), and ACNHRLASC and ACSGEERAC (from the ACX_6_C library) in the paper of Zygiel and co-workers [17]. Such ancestral mutations represent library construction artifacts, which seem to be unavoidable and might happen in the construction of many combinatorial phage display libraries. These mutant clones are nonspecifically enriched during biopanning in a target-independent manner associated with amplification bias. This opinion is in accordance with previous reports on the existence of ancestral point mutations in fd-tet (used to make f88-4, fUSE5, and f3-15mer libraries) phage display peptide libraries [7] and propagation-related parasitic enrichment of ancestral indel mutations in a focused M13 phage protein library [36]. Based on the second scenario, the M13KE genome is stable enough to be used for the construction and selection cycles of phage display libraries. 

## 4. Materials and Methods

### 4.1. Phage Display Library and Biopanning

The phage vector M13KE used in Ph.D.^TM^-7 Phage Display Peptide Library (New England Biolabs, Ipswich, MA, USA) displays random heptapeptides fused to the N-terminus of the phage minor coat protein pIII. Each peptide is expressed in a pentavalent manner on the surface of five pIII proteins. This library was used for panning on recombinant mouse CD4 protein (Sino Biological, China) as described previously [37] with some modifications. After three rounds of library selection, bound phages were recovered with acidic glycine (0.2 M glycine-HCl, pH = 2.2, 1 mg/mL BSA) and then neutralized with 1 M Tris-HCl (pH = 9.1). The recovered phages were plated on IPTG-X-gal agar plates and a number of plaques were picked out for Sanger sequencing. 

### 4.2. Phage Amplification and Extraction 

Escherichia coli strain ER2738 was used to amplify phage virions. The phage pool was cultured in 20 mL early log ER2738 bacterial cells (5 h, 37 °C, 250 rpm). After removing bacterial cells and debris, the amplified library was purified by adding PEG/NaCl buffer (1/6 volume of 20% PEG/2.5 M NaCl, overnight incubation at 4 °C) and centrifugation (12,000× *g*, 10 min, 4 °C).

### 4.3. Phage Titration 

Double agar overlay method was used to determine the titer of phage suspensions for competitive propagation assay. For this purpose, phage suspensions were serially diluted (10-fold) in LB medium and incubated with a mid-log phase ER2738 culture (300 µL, OD_600_ = 0.5) for 5 min at room temperature. The infected cells were then transferred into 3mL pre-heated top agar (0.7%), plated onto LB agar/Tet/X-gal/IPTG medium, and incubated overnight at 37 °C before counting plaques. Each sample was plated in triplicate and titer was determined from plates with an even distribution of plaques. 

To determine the titer of phage suspensions for ELISA, quantitative PCR (qPCR) was used as described elsewhere [38] with minor modifications. M13KE double-stranded DNA (dsDNA) (New England Biolabs, Ispswich, MA, USA) was used to plot the calibration curve and quantify phage titer. During PCR, phage ssDNA is converted into dsDNA and SYBR Green stains dsDNA. Eight 10-fold dilutions of purified phage suspensions were serially prepared and incubated with 10 U of DNAase I (Thermo Fisher Scientific, Waltham, MA, USA) at 37 °C for 1 h, to remove residual DNA that is not from intact phage particles, followed by enzyme inactivation at 95 °C for 15 min. A 20 µL PCR reaction was run using Agilent Mx3005P QPCR System in which each reaction well contained 7.8 µL of Applied Biosystems^TM^ Power SYBR^TM^ Green 2X Master Mix (Themo Fisher Scientific, Waltham, MA, USA), 2 µL of denatured phage DNA, 0.1 µL of each of the primers (500 nM), and 7.8 µL of RNase-free dH_2_O. The kit contains highly purified Applied Biosystems^TM^ AmpliTaq Gold^TM^ DNA polymerase, LD. This polymerase is a chemically modified hot start PCR enzyme. Forward (5′-CACCGTTCATCTGTCCTCTTT-3′) and reverse (5′-CGACCTGCTCCATGTTACTTAG-3′) primers bind specifically to the genome of M13KE at positions 1025–1045 and 1099–1120, respectively [38]. Two temperature PCR was applied, and PCR cyclic conditions were as follows: 95 °C for 10 min, 40 cycles of 95 °C for 15 s and 60 °C for 1 min by a melt curve setting of 95 °C for 1 min and ramp from 55–95 °C (30 s). Phage quantification was conducted by comparing the observed cycle threshold (Ct) values of sample with standard curve. All reactions were performed in triplicate. To convert the concentration of DNA into genome copies per microliter (gc/µL), the following formula was used, and dilution factor was included for concentration calculations [38]:

[genome copies (gc)/µL] = [dsDNA g/µL]/[DNA size (bp) × 607.4 + 157.9] × (6.02 × 10^23^)

### 4.4. Sanger Sequencing of White Plaques 

A number of white plaques were picked out for sequencing and used as template in PCR (plaque or whole phage PCR) conducted by a high-fidelity DNA polymerase. A part of phage genome was amplified in a 50 µL PCR reaction containing 25 µL of Q5 High-Fidelity 2X Master Mix (New England Biolabs, Ipswich, MA, USA), 2.5 µL of each of the forward and reverse primers (10 µM), 1 µL of water-diluted plaque, and 19 µL of nuclease-free water. Primers were designed using SnapGene software (from Insightful Science; available at snapgene.com) and the sequences of primers were 5′-TAGTCCTCAAAGCCTCTGTAGC-3′(forward) and 5′-CAATAGGAACCCATGTACCG-3′(reverse). The following cyclic conditions were used: initial denaturation at 98 °C for 30 s, 30 cycles of denaturation at 98 °C for 10 s, annealing at 53 °C for 20 s, and extension at 72 °C for 20 s. The final extension was performed at 72 °C for 2 min. The amplified DNA was purified by QIAquick PCR purification kit (Qiagen, Hilden, Germany) and subjected to sequencing by ABI sequencer (Eurofins Genomics, Ebersberg, Germany).

### 4.5. Phage ELISA

Recombinant CD4 (0,5 µg/well) was immobilized on a 96-well Maxisorp plate (Nunc, Thermo Fisher) at 4 °C overnight followed by addition of 100 µL of blocking buffer (5% BSA dissolved in PBS) at 37 °C for 3 h. After two washes with 250 µL of PBST (0.1% tween 20), pre-blocked (5% BSA, 37 °C, 1 h) phages (10^10^ pfu/well) were added and incubated for 0.5 h with shaking. Unbound phages were removed by 5 consecutive washes and bound phages were incubated with 100 µL of HRP-conjugated anti-M13 antibody (Sino Biological, Beijing, China). Samples were incubated with 100 µL of 3,3′,5,5′-tetramethylbenzidine (Merck, Darmstadt, Germany) in the dark until color development (15 min) and the reaction was terminated with 100 µL of 2M H_2_SO_4_. The absorbance was measured on a FLUOstar^®^ Omega reader (BMG Labtech, Ortenberg, Germany) at 450 nm and 540 nm (as reference wavelength).

### 4.6. Whole Genome Sequencing of White Plaque

The double-stranded replicative form (RF) DNA of WSLGYTG-displaying phage clone producing white plaques was purified using the NucleoSpin^®^ plasmid kit (Macherey-Nagel, Düren, Germany) according to the manufacturer’s protocol. Whole genome sequencing (WGS) was performed on the purified DNA using Illumina NovaSeq 6000 platform with a PE150 base pair (bp) strategy (Novogene, Cambridge, UK).

### 4.7. Plaque Count-and NGS-Based Competitive Propagation Assay (CPA)

An equal number (1 × 10^9^ pfu) of white-Ph-WSLGYTG clone and Ph.D.^TM^-7 peptide library was amplified (250 rpm, 37 °C) in a 20 mL early log ER2738 culture in LB for 4.5 h. At incubation times of 0, 150 and 270 min, samples were withdrawn and spun (4000 g) at 4 °C for 10 min. To determine the titer of white and blue plaques in the culture, 10 µL of phage supernatant was serially diluted (10-fold dilutions) and plated using 200 µL of ER2738 strain (OD_600_ = 0, 5) and 3 mL top agar (0.7%). Plaques were assessed as either white or blue and counted on plates with 50–300 plaques. 

The remaining 2,8 mL phage supernatant was used for DNA extraction using NucleoSpin ^®^ Plasmid kit for isolation of M13 DNA (Macherey-Nagel, Düren, Germany). DNA was eluted in 10 µL of RNase-free water and its concentration and purity was measured using NanoDrop^TM^. PCR was run on eluted phage DNA using Q5 High-Fidelity 2X Master Mix (New England Biolabs, Ipswich, MA, USA) in 50 µL reaction volumes containing 25 µL of master mix, 5 µL of phage DNA, and 2.5 µL (10 μM) of each of the forward and reverse primers. The primers contained phage genome-specific sequences and Illumina-compatible sequencing adaptors for subsequent NGS analysis. The sequences of primers are as follows [39]: 

Forward: 5′-AATGATACGGCGACCACCGAGATCTACACTTCCTTTAGTGGTACCTTTCTATTCTC٭A

Reverse: 5′-CAAGCAGAAGACGGCATACGAGATCGGTCTATGGGATTTTGCTAAACAACTTT٭C 

PCR was performed as follows: initial denaturation at 98 °C for 30 s, and 20 cycles of denaturation at 98 °C for 10 s, annealing at 60 °C for 30 s, and extension at 72 °C for 20 s followed by final extension at 72 °C for 2 min. The correct size of PCR product was evaluated on 1% TAE agarose gel. The PCR product was purified with QIAquick PCR purification kit (Qiagen, Düren, Germany) and used for NGS analysis.

Illumina next-generation sequencing was performed by Center for Genomic Medicine, Copenhagen University Hospital, Rigshospitalet (Copenhagen, Denmark) using the MiSeq platform and the MiSeq v2 Nano reagent kit. Each purified PCR product obtained from the previous step was run individually on an Illumina flow cell and subjected to amplification. Single-end sequencing strategy was conducted for 53 cycles including a custom sequencing primer (ACACTTCCTTTAGTGGTACCT TTC TAT TCT CAC TC٭T). Asterisks in the sequences of primers refer to the presence of phosphorothioate. 

### 4.8. Analysis of Illumina NGS Data

The resulting FASTQ file was uploaded into a MATLAB script [40] with some modifications. Data analysis comprises different steps including read filtering, translation of nucleotides into amino acids, and calculation of frequencies for the identified hits. The script recognizes the sequenced nucleotides and translates them into amino acids. The variable region of phage genome contains the sequences of 7-amino acid displayed peptide, (consisting of 21 nucleotides) and GGGS linker (12 nucleotides) at the C-terminus of displayed peptide. Reads with invalid amino acids (containing ‘*’) within the displayed peptide or not containing a complete and correct linker sequence were removed (specified as total number of removed reads in Table 2). Thereafter, 95.2%, 96.8%, and 97.9% of reads remained for time points 0, 150, and 270, respectively. The remaining reads (specified as total number of clean reads in Table 2) were sorted according to their frequencies. The relative abundance of WSLGYTG clone was calculated as percentage based on the total number of clean reads at different time points. The script used for data analysis can be found in Supplementary Materials.

## 5. Conclusions

Due to the growing popularity of phage display for peptide discovery, commercial phage display libraries have gained considerable attention for the discovery of translational peptides. Since M13KE-based Ph.D. libraries are extensively used in phage display research, identifying the origin of white plaques observed upon working with these libraries is crucial. Our findings provide clear evidence for the fact that white clones might derive from the library, and not every white plaque should be viewed as environmental contamination. In the case of endogenous origin of white clones, all manufacturer’s recommendations to remove these plaques from biopanned phage pools, such as sterilization of equipment and reagents, are not helpful. Upon finding white plaques during library selection, we highly recommend determining their genetic identity. This genotypic characterization can be easily conducted by picking out a number of white plaques and sequencing them. Sequencing can be performed by using (−96 gIII and −28 gIII primers) provided in the Ph.D.^TM^-7 library kit or any type of custom-made primers, which target genomic regions able to show nucleotide differences between environment-derived WT-M13 and library-derived M13KE. Since mutations giving rise to propagation advantage may prevent the enrichment of true target binding clones, this work highly emphasizes the importance of identifying and genetically characterizing these mutants to avoid their misidentification as target-specific binders. 

## Figures and Tables

**Table 1:** White/blue plaque count-based titration in competitive propagation assay. The increased titer of white plaques is significantly higher than blue plaques during amplification period. Ratio was obtained through dividing the titer of white plaques by the titer of blue plaques at each time point. Percent is calculated by dividing the titer of white plaque by the titer of both white and blue plaques multiplied by 100.

Incubation Time	White Plaque Titer (pfu/mL)	Blue Plaque Titer (pfu/mL)	White/Blue Ratio	Percent of White Plaques in the Phage Pool
t = 0	4.9 × 10^7^	4.1 × 10^7^	1.2	54.4%
t = 150	5.3 × 10^8^	8.0 × 10^7^	6.6	86.9%
t = 270	2.7 × 10^10^	1.9 × 10^8^	14.3	99.3%

**Table 2:** Number of NGS reads at different time points. The table shows the total number of reads obtained after NGS sequencing, the number of clean reads used for data analysis, and the number of removed reads after filtering.

Incubation Time	Total Number of Reads	Total Number of Clean Reads	Total Number of Removed Reads	Percent of Removed Reads
t = 0	879,645	837,278	42,367	4.8
t = 150	1,141,980	1,105,681	36,299	3.2
t = 270	1,261,717	1,235,481	26,236	2.1

## Data Availability

Not applicable.

## Supplementary Materials

The following supporting information can be downloaded at: https://www.mdpi.com/article/10.3390/ijms23063308/s1.

## Abbreviations

Biopanning data bank (BDB), enzyme linked immunosorbent assay (ELISA), horseradish peroxidase (HRP), isopropyl β-D-1- thiogalactopyranoside (IPTG), mouse CD4 (mCD4), New England Biolabs (NEB), phosphate-buffered saline (PBS), phosphate-buffered saline, 0.1% Tween ^®^ (PBST), polyethylene glycol (PEG), plaque forming unit (pfu), propagation-related target-unrelated peptide (Pr-TUP), replicative form (RF), Scanner and Reporter of Target-Unrelated Peptides (SAROTUP), Selection-related target-unrelated peptide (Sr-TUP), Tris-acetate-EDTA (TAE), tetracycline (Tet), 3,3′,5,5′-tetramethylbenzidine (TMB), target-related peptide (TRP), whole genome sequencing (WGS), wild-type environmental M13 (WT-M13), library clone not expressing any peptide (WT-M13KE), 5-bromo-4-chloro-3-indolyl-β-d-galactopyranoside (X-gal)

## References

[1] Smith G.P. (1985). Filamentous Fusion Phage: Novel Expression Vectors That Display Cloned Antigens on the Virion Surface. Science.

[2] Smith G.P. (2019). Phage Display: Simple Evolution in a Petri Dish (Nobel Lecture). Angew. Chem. Int. Ed..

[3] (2020). Instruction Manual Ph.D. Phage Display Libraries. no. 5.0. https://international.neb.com/-/media/nebus/files/manuals/manuale8100_e8101_e8110_e8111_e8120.pdf?rev=0ec88f21c8e5411a9959f3db8f360b7b&hash=9974F2C3DA3C67E92A18139069D74F4E.

[4] Noren K.A., Noren C.J. (2001). Construction of High-Complexity Combinatorial Phage Display Peptide Libraries. Methods.

[5] Pande J., Szewczyk M.M., Grover A.K. (2010). Phage Display: Concept, Innovations, Applications and Future. Biotechnol. Adv..

[6] Menendez A., Jamie K.S. (2005). The Nature of Target-Unrelated Peptides Recovered in the Screening of Phage-Displayed Random Peptide Libraries with Antibodies. Anal. Biochem..

[7] Thomas W.D., Golomb M., Smith G.P. (2010). Corruption of Phage Display Libraries by Target-Unrelated Clones: Diagnosis and Countermeasures. Anal. Biochem..

[8] Brammer L.A., Bolduc B., Jessica, Kass L., Kristin, Felice M., Christopher, Noren J., Hall M.F. (2008). A Target-Unrelated Peptide in an M13 Phage Display Library Traced to an Advantageous Mutation in the Gene II Ribosome-Binding Site. Anal. Biochem..

[9] Nguyen K.T., Adamkiewicz M.A., Hebert L.E., Zygiel E.M., Boyle H.R., Martone C.M., Meléndez-Ríos C.B., Noren K.A., Noren C.J., Hall M.F. (2014). Identification and Characterization of Mutant Clones with Enhanced Propagation Rates from Phage-Displayed Peptide Libraries. Anal. Biochem..

[10] He B., Chen H., Li N., Huang J. (2019). Sarotup: A Suite of Tools for Finding Potential Target-Unrelated Peptides from Phage Display Data. Int. J. Biol. Sci..

[11] He B., Jiang L., Duan Y., Chai G., Fang Y., Kang J., Yu M., Li N., Tang Z., Yao P. (2018). Biopanning Data Bank 2018: Hugging Next Generation Phage Display. Database.

[12] Cloning Vector M13KE, Complete Sequence New England Biolabs. https://international.neb.com/-/media/nebus/page-images/tools-and-resources/interactive-tools/dna-sequences-and-maps/text-documents/m13kegbk.txt?rev=9ee51a577a21478ab4c9b11957e89282&hash=C39F8D6CDB63483CA2F8E79E28C08EDF.

[13] Brown K.C. (2010). Peptidic Tumor Targeting Agents: The Road from Phage Display Peptide Selections to Clinical Applications. Curr. Pharm. Des..

[14] Smith G.P., Scott J.K. (1993). Libraries of Peptides and Proteins Displayed on Filamentous Phage. Methods in Enzymology.

[15] Ahmadi A., Ayyadevara V.S.S.A., Baudry J., Roh K.-H. (2021). Calcium Signaling on Jurkat T Cells Induced by Microbeads Coated with Novel Peptide Ligands Specific to Human Cd3ε. J. Mater. Chem. B.

[16] Ionov Y., Rogovskyy A.S. (2020). Comparison of Motif-Based and Whole-Unique-Sequence-Based Analyses of Phage Display Library Datasets Generated by Biopanning of Anti-Borrelia Burgdorferi Immune Sera. PLoS ONE.

[17] Zygiel E.M., Noren K.A., Adamkiewicz M.A., Aprile R.J., Bowditch H.K., Carroll C.L., Cerezo M.A.S., Dagher A.M., Hebert C.R., Hebert L.E. (2017). Various Mutations Compensate for a Deleterious lacZα Insert in the Replication Enhancer of M13 Bacteriophage. PLoS ONE.

[18] Dotto G.P., Horiuchi K., Zinder N.D. (1984). The Functional Origin of Bacteriophage F1 DNA Replication: Its Signals and Domains. J. Mol. Biol..

[19] Johnston S., Ray D.S. (1984). Interference between M13 and oriM13 Plasmids Is Mediated by a Replication Enhancer Sequence near the Viral Strand Origin. J. Mol. Biol..

[20] Messing J., Gronenborn B., Müller-Hill B., Hopschneider P.H. (1977). Filamentous Coliphage M13 as a Cloning Vehicle: Insertion of a HindII Fragment of the Lac Regulatory Region in M13 Replicative Form in Vitro. Proc. Natl. Acad. Sci. USA.

[21] Rakonjac J., Bennett N.J., Spagnuolo J., Gagic D., Russel M. (2011). Filamentous Bacteriophage: Biology, Phage Display and Nanotechnology Applications. Curr. Issues Mol. Biol..

[22] Marvin D.A. (1998). Filamentous Phage Structure, Infection and Assembly. Curr. Opin. Struct. Biol..

[23] Marvin D.A., Symmons M.F., Straus S.K. (2014). Structure and Assembly of Filamentous Bacteriophages. Prog. Biophys. Mol. Biol..

[24] Straus S.K., Scott W.R.P., Symmons M.F., Marvin D.A. (2008). On the Structures of Filamentous Bacteriophage Ff (Fd, F1, M13). Eur. Biophys. J..

[25] Day L.A., Marzee C.J., Reisberg S.A., Casadevall A. (1988). DNA Packing in Filamentous Bacteriophages. Annu. Rev. Biophys. Biophys. Chem..

[26] Specthrie L., Bullitt E., Horiuchi K., Model P., Russel M., Makowski L. (1992). Construction of a Microphage Variant of Filamentous Bacteriophage. J. Mol. Biol..

[27] Marvin D.A. (1966). X-Ray Diffraction and Electron Microscope Studies on the Structure of the Small Filamentous Bacteriophage Fd. J. Mol. Biol..

[28] Linderoth N.A., Simon M.N., Russel M. (1997). The Filamentous Phage pIV Multimer Visualized by Scanning Transmission Electron Microscopy. Science.

[29] Opalka N., Beckmann R., Boisset N., Simon M.N., Russel M., Darst S.A. (2003). Structure of the Filamentous Phage pIV Multimer by Cryo-Electron Microscopy. J. Mol. Biol..

[30] Wang Y.A., Yu X., Overman S., Tsuboi M., Thomas G.J., Egelman E.H. (2006). The Structure of a Filamentous Bacteriophage. J. Mol. Biol..

[31] Sattar S., Bennett N.J., Wen W.X., Guthrie J.M., Blackwell L.F., Conway J.F., Rakonjac J. (2015). Ff-Nano, Short Functionalized Nanorods Derived from Ff (F1, Fd, or M13) Filamentous Bacteriophage. Front. Microbiol..

[32] Xu J., Dayan N., Goldbourt A., Xiang Y. (2019). Cryo-Electron Microscopy Structure of the Filamentous Bacteriophage IKe. Proc. Natl. Acad. Sci. USA.

[33] Conners R., McLaren M., Łapińska U., Sanders K., Stone M., Blaskovich M.A.T., Pagliara S., Daum B., Rakonjac J., Gold V.A.M. (2021). CryoEM Structure of the Outer Membrane Secretin Channel pIV from the F1 Filamentous Bacteriophage. Nat. Commun..

[34] He B., Chen H., Huang J. (2019). PhD7Faster 2.0: Predicting Clones Propagating Faster from the Ph.D.-7 Phage Display Library by Coupling PseAAC and Tripeptide Composition. PeerJ.

[35] Peris J.B., Davis P., Cuevas J.M., Nebot M.R., Sanjuán R. (2010). Distribution of Fitness Effects Caused by Single-Nucleotide Substitutions in Bacteriophage F1. Genetics.

[36] Plessers S., van Deuren V., Lavigne R., Robben J. (2021). High-Throughput Sequencing of Phage Display Libraries Reveals Parasitic Enrichment of Indel Mutants Caused by Amplification Bias. Int. J. Mol. Sci..

[37] Dai X., Cai C., Xiao F., Xiong Y., Huang Y., Zhang Q., Xiang Q., Lou G., Lian M., Su Z. (2014). Identification of a Novel aFGF-Binding Peptide with Anti-Tumor Effect on Breast Cancer from Phage Display Library. Biochem. Biophys. Res. Commun..

[38] Peng X., Nguyen A., Ghosh D. (2018). Quantification of M13 and T7 Bacteriophages by TaqMan and SYBR Green qPCR. J. Virol. Methods.

[39] AC’t Hoen P., Jirka S.M., Bradley R., Schultes E.A., Aguilera B., Pang K.H., Heemskerk H., Aartsma-Rus A., van Ommen G.J., den Dunnen J.T. (2012). Phage Display Screening without Repetitious Selection Rounds. Anal. Biochem..

[40] Brinton L.T., Bauknight D.K., Dasa S.S.K., Kelly K.A. (2016). PHASTpep: Analysis Software for Discovery of Cell-Selective Peptides Via Phage Display and Next-Generation Sequencing. PLoS ONE.

