# Supplementary material for: A White Plaque, Associated with Genomic Deletion, Derived from M13KE-Based Peptide Library Is Enriched in a Target-Unrelated Manner during Phage Display Biopanning Due to Propagation Advantage
Source: Int J Mol Sci. 2022 Mar 18;23(6):3308. doi: 10.3390/ijms23063308 (PMC8950111; doi:10.3390/ijms23063308)
Supplement: Supplementary file 1 [file ijms-23-03308-s001.zip › ijms-1621625-supplementary.pdf]

```
function [AA_Clean, AA_Removed] = Load_NGS_Data_Long_Sequence(FileName,
Library, WriteToExcel)

    %%% Library chosen decides length of sequences to load %%%

    if strcmp(Library, 'PhD7')
        Seq_Length = 7 + 4;
        Seq_Nucleotides = [1:33];
    elseif strcmp(Library, 'PhD12')
        Seq_Length = 12 + 4;
        Seq_Nucleotides = [161:209] - 33;
    elseif strcmp(Library, 'PhDC7C')
        Seq_Length = 3 + 7 + 4;
        Seq_Nucleotides = [161:203] - 33;
    else
        error('Use either PhD7, PhD12, or PhDC7C.');
    end

    %%% Read the sequences from the fastq file %%%
    disp('Reading in fastq file');
    [~, Seqs, ~] = fastqread(FileName);

    %%% Removing flanking region
    disp('Removing flanking regions');
    Seqs_Nucleotide = cell(length(Seqs), 1);
    Seqs_Error_Length = cell(length(Seqs), 1);

    for i = 1 : length(Seqs)

        if length(Seqs{i}) >= Seq_Nucleotides(end)
            Seqs_Nucleotide{i} = Seqs{i}(Seq_Nucleotides);
        else
            Seqs_Error_Length{i} = Seqs{i};
        end

    end

    Seqs_Nucleotide = Seqs_Nucleotide(~cellfun('isempty', Seqs_Nucleotide));
    Seqs_Error_Length = Seqs_Error_Length(~cellfun('isempty',
Seqs_Error_Length));

    %%% Convert nucleotides to AA and put into a frequency sorted matrix %%%
    disp('Conversion to amino acids');
    AA_Array = nt2aa(Seqs_Nucleotide,...
        'AlternativeStartCodons',false,...
        'ACGTonly', false);

    disp('Tabulating amino acids');
    AA_Cell = tabulate(AA_Array);
```

```
%%%% Clean up data by ensuring proper end and lack of '*' %%%%%
disp('Cleaning up the sequences.');

%Remove invalid '*' sequences
disp('Removing sequences with *.');
InvIdx1 = strfind(AA_Cell(:, 1), '*');

AA_Removed = AA_Cell(~cellfun('isempty',InvIdx1),:);
AA_Clean = AA_Cell(cellfun('isempty',InvIdx1),:);

%Ensure proper ending, i.e., 'GGGS'
disp('Ensuring proper ending of GGGS.');
InvIdx2 = strfind(AA_Clean(:, 1), 'GGGS');
InvIdx2 = cellfun(@(InvIdx2) find(InvIdx2 == Seq_Length - 3),...
    InvIdx2, 'UniformOutput', false);

AA_Removed = [AA_Removed; AA_Clean(cellfun('isempty', InvIdx2), :)];
AA_Clean = AA_Clean(~cellfun('isempty', InvIdx2), :);


if strcmp(Library, 'PhDC7C')

    %Need to check for 'AC' beginning
    InvIdx3 = strfind(AA_Clean(:, 1), 'AC');
    InvIdx3 = cellfun(@(InvIdx3) find(InvIdx3 == 1),...
        InvIdx3, 'UniformOutput', false);

    AA_Removed = [AA_Removed; AA_Clean(cellfun('isempty', InvIdx3), :)];
    AA_Clean = AA_Clean(~cellfun('isempty', InvIdx3), :);

    %and 'C' after sequence
    InvIdx4 = strfind(AA_Clean(:, 1), 'C');
    InvIdx4 = cellfun(@(InvIdx4) find(InvIdx4 == 10),...
        InvIdx4, 'UniformOutput', false);

    AA_Removed = [AA_Removed; AA_Clean(cellfun('isempty', InvIdx4), :)];
    AA_Clean = AA_Clean(~cellfun('isempty', InvIdx4), :);

end


%Sort the cells
AA_Clean = sortrows(AA_Clean, -2);
AA_Removed = sortrows(AA_Removed, -2);

%Update relative percentage for clean and removed lists
AA_Clean(:, 3) = num2cell(100 * cell2mat(AA_Clean(:, 2)) /...
    sum(cell2mat(AA_Clean(:, 2))));
AA_Removed(:, 3) = num2cell(100 * cell2mat(AA_Removed(:, 2)) /...
```

```
        sum(cell2mat(AA_Removed(:, 2))));


    %%% Decide upon writing to an excel file %%%
    if ~strcmp(WriteToExcel, 'No')
        %Limit set to 100,000 sequences - can be increased to max 8,640,909
        Seq_Limit = 1:10^3;

        AA_Clean_Table = table(AA_Clean(Seq_Limit,1),...
                               cell2mat(AA_Clean(Seq_Limit,2)),...
                               cell2mat(AA_Clean(Seq_Limit,3)),...
                           'VariableNames', {'AA', 'Number', 'Relative (%)'});
        AA_Removed_Table = table(AA_Removed(Seq_Limit,1),...
                               cell2mat(AA_Removed(Seq_Limit,2)),...
                               cell2mat(AA_Removed(Seq_Limit,3)),...
                           'VariableNames', {'AA', 'Number', 'Relative (%)'});

        writetable(AA_Clean_Table, WriteToExcel + "_AA_Clean.xls");
        writetable(AA_Removed_Table, WriteToExcel + "_AA_Removed.xls");
    end

end
```
